# Epidemiology of hypertension among patients with type 2 diabetes in the Democratic Republic of Congo

**DOI:** 10.4102/phcfm.v17i1.4712

**Published:** 2025-03-20

**Authors:** Xoliswa Simelane, Jean-Pierre Fina-Lubaki, Joel M. Francis

**Affiliations:** 1 Division of Epidemiology and Biostatistics, Faculty of Health Sciences, University of the Witwatersrand, Johannesburg, South Africa; 2 Department of Family Medicine and Primary Health Care, Faculty of Medicine, Protestant University of Congo, Kinshasa, Democratic Republic of Congo; 3 Department of Family Medicine and Primary Health Care, Faculty of Health Science, University of the Witwatersrand, Johannesburg, South Africa

**Keywords:** type 2 diabetes, hypertension, epidemiology, urban setting, sub-Saharan Africa

## Abstract

**Background:**

Hypertension is a common comorbidity among patients with type 2 diabetes (T2D) and is associated with poorer treatment outcomes.

**Aim:**

To describe the epidemiology of hypertension among patients with T2D in Kinshasa, Democratic Republic of the Congo.

**Setting:**

A multisite study among 20 randomly selected health facilities in Kinshasa.

**Methods:**

This was an analytical cross-sectional study among 620 participants with T2D. The overall prevalence of hypertension and uncontrolled hypertension was determined. Multivariable mixed effects logistic regression determined factors associated with hypertension and hypertension control among participants with T2D.

**Results:**

One-third (34.7%) of study participants were classified as having hypertension. The factors associated with hypertension were unemployment (adjusted odds ratio [aOR] = 1.93, 95% confidence interval [CI]: 1.18–3.17), overweight (aOR = 2.71; 95% CI: 1.78–4.13), diabetes duration ≥ 5 years (aOR = 1.84, 95% CI: 1.24–2.73), protestant religion (aOR = 0.48, 95% CI: 0.29–0.82) and severe diabetes distress (aOR = 0.47; 95% CI: 0.28–0.79). The prevalence of uncontrolled hypertension among participants with diabetes-hypertension comorbidity was 50.2%. Being overweight was associated with uncontrolled hypertension (aOR = 2.02; 95% CI: 1.08–3.79).

**Conclusion:**

Hypertension was common among patients with T2D in Kinshasa, Democratic Republic of Congo (DRC), and in most patients it was uncontrolled. There is a need to strengthen the hypertension prevention and control strategies among patients with T2D, including lifestyle modifications to maintain optimal body weight.

**Contribution:**

This study provides insight into the diabetes-hypertension comorbidity in an African urban setting.

## Introduction

Type 2 diabetes (T2D) is a major global health challenge,^1^ with an estimated 462 million individuals affected worldwide, representing over 90% of all diabetes cases.^2^ An alarming 45% of people have undiagnosed diabetes, which is overwhelmingly type 2.^1^ The prevalence of T2D is unevenly distributed across regions, with low- and middle-income countries experiencing the most significant increases.^2^ This prevalence, while lower than in some other regions, is rapidly increasing because of urbanisation, lifestyle changes and limited access to healthcare services.^2^ In sub-Saharan Africa (SSA), the number of adults with T2D is expected to more than double by 2045, reaching 55 million.^2,3^ The Democratic Republic of Congo (DRC) faces a significant public health challenge with T2D,^4^ affecting approximately 4.8% of the adult population.^5^

Type 2 diabetes is frequently accompanied by various comorbidities such as renal disease,^6^ hypertension^7^ and chronic obstructive pulmonary disease,^8^ which exacerbate the disease burden and complicate management. Among these comorbidities, hypertension is one of the most prevalent. A meta-analysis conducted by Uthman and colleagues found that the prevalence of hypertension in individuals with T2D ranged from 30% to 90% globally.^9^ Similarly, studies in the SSA have reported prevalence of hypertension among type 2 diabetes mellitus (T2DM) as high as 58.1%: Central Africa had the highest prevalence of 77.6%, Southern Africa 69.1%, West Africa 51.5% and East Africa 53.0%.^10^ A systematic review conducted in Ethiopia found a pooled prevalence of hypertension to be 55%, with higher prevalence among urban residents.^11^ A study in the province of Kivu, DRC, found a prevalence of hypertension of 59.6% among participants with T2D.^12^

This comorbidity is influenced by multiple factors, including increasing age, overweight, physical inactivity, dietary habits, employed, longer duration of diabetes, male sex and urban residence.^10^ Obesity is a significant risk factor for both conditions.^7,13^ In the previous cited study in Kivu, DRC, the authors found that hypertension was associated with overweight and chronic kidney disease.^12^ In the context of T2DM, hypertension accelerates the progression of diabetic complications, including nephropathy, retinopathy and neuropathy.^12^ Addressing these issues is crucial as the combined effect of T2D and hypertension significantly increases morbidity and mortality.^1^

In SSA, as in the DRC, recent studies have shown that less than one-third of persons with diabetes reached glycaemic targets in SSA and in Kinshasa.^14,15^ Prevalence of uncontrolled diabetes in Kinshasa was reported to be 67.6% among T2DM patients.^15^ The co-existence of hypertension and T2D amplifies the risk of adverse health outcomes, making it imperative to address these conditions concomitantly.^9,13^

Uncontrolled hypertension remains a significant challenge among T2DM patients.^16,17,18,19^ Studies have shown that despite the availability of antihypertensive medications, a large proportion of T2DM patients with hypertension fail to achieve optimal blood pressure control.^19,20^ A systematic review conducted in SSA found the pooled prevalence of uncontrolled hypertension among T2DM patients to be 74.5%.^19^ Factors contributing to uncontrolled hypertension include poor medication adherence, inadequate healthcare access and a lack of patient education on lifestyle modifications.^9,16,17^

Effective management of these conditions can prevent complications, improve quality of life and reduce healthcare costs.^1,2,21^ For example, integrated care models that focus on both T2D and hypertension have been shown to improve patient outcomes and reduce hospital admissions.^22^

Several studies have reported on hypertension,^7,10,11,12,13^ or uncontrolled blood pressure^9,16,17^ among patients with T2D in SSA, but none of the studies were conducted in Kinshasa, DRC.

This study aimed to investigate the epidemiology of hypertension, and of uncontrolled hypertension, among T2D patients in Kinshasa, DRC.

The findings of this study would inform the need for interventions to improve hypertension prevention and optimal treatment outcome among patients with T2D.

## Research methods and design

### Study design

This study was an analytical cross-sectional survey of patients with T2D from Kinshasa, DRC.

### Setting

The study was multisite within Kinshasa, which is the capital of the DRC. It has a population of 17 032 322 inhabitants spread over an area of 9965 km^2^, representing a density of 13 662 per/km^2^.^23^ As in other parts of the DRC, the population is growing fast because of rural exodus. The employment is high among the population who rely on informal activities for surviving.^24^ Diabetes care in Kinshasa is mainly organised by Kinshasa Primary Care Network, an organisation that reunited state and private organisations.^25^ This organisation owns a total of 66 health facilities (1 referral hospital and 65 health centres) distributed across 24 health districts. Participants were recruited from 20 randomly selected health facilities; the study protocol was previously published.^26^

### Study population and sampling strategy

The study population consisted of persons with T2D attending Kinshasa Primary Care Network for diabetes care. There were 7326 patients with T2D registered in 2020. The inclusion criteria were age ≥ 18 years, receiving diabetes treatment for at least 6 months and consenting to the study. Exclusion criteria were pregnancy and having difficulty in communicating. For this study, all participants in the dataset were included in the analysis. Given the varying numbers of patients with diabetes across these facilities, sampling with probability proportional to size method was used. In the first stage, 20 healthcare facilities were randomly chosen from a total of 48. In order to increase the probability of individuals in the population to enter in the study, the same number of individuals were sampled from each selected health centre. Thus, 31 participants with T2D were systematically sampled from each of the chosen healthcare facilities.^26^

### Data collection

The data were collected with questionnaires designed on REDCap,^27^ using a tablet or smartphone by the principal researchers assisted by three medical doctors. The questionnaire was administered in Lingala or French following the preference of the participant. The administration of the questionnaire lasted approximately 60 min. At the end, 2 mL of venous blood were drawn from the participants for a glycosylated haemoglobin assay. The data collection took place between November 2021 and September 2022 in Kinshasa, DRC.

### Variables

#### Outcome variables

In this study, two outcomes of interest were considered: the presence of hypertension and control of blood pressure. The presence of hypertension was defined as the existence of hypertension in the medical history of the participant. In our setting, hypertension is defined as a systolic blood pressure level of 140 mmHg or higher and/or a diastolic blood pressure level of 90 mmHg or higher,^28^ measured on three different occasions. The information was sought in the medical booklet of the participants.

The blood pressure was considered as uncontrolled with systolic blood pressure of 130 mmHg or higher and/or a diastolic blood pressure of 80 mmHg or higher^28^; otherwise, when the systolic blood pressure was below 130 mmHg and/or the diastolic blood pressure was below 80 mmHg, the blood pressure was considered as controlled. The blood pressure was measured for all the participants while seated using an EDAN automatic sphygmomanometer three times separated by a 5-min interval. The mean was calculated based on the three readings.

#### Exposure variables

The exposure variables were sociodemographic, clinical and psychological.

*Sociodemographic parameters*: A distance from the participant’s place of residence to the health centre of ˂ 5 km or ˂ 1 h was defined as less than 5 km, while ˃ 5 km or > 1 h was defined as 5 km or more, in line with the health system norms in the DRC and as illustrated in the study by Kaboru and Namegabe.^29^ Poverty was defined as daily income of < 3800 Congolese Francs (< $1.9) set as the threshold for poverty.^30^

*Clinical parameters*: The body mass index (BMI) was calculated as weight (kg)/height (square metres) and was categorised as follows: underweight (BMI < 18.5 kg/m^2^), normal weight (18.5 kg/m^2^ – 24.99 kg/m^2^), overweight (BMI ≥ 25 kg/m^2^) and obese ≥ 30 kg/m^2^.^31^ With reference to the study by Wake et al. in Ethiopia,^32^ the duration of diabetes was categorised into two: less than 5 years and 5 years and more. We considered three options for treatment regimens: insulin alone, oral hypoglycaemic drugs and mixed treatment (insulin plus oral hypoglycaemic drugs). Hypertension treatment into two categories: ‘no’ for those without treatment and ‘yes’ for those taking treatment. Poly treatment was categorised into two: ‘no’ and ‘yes’; ‘no’ was a treatment with less than five prescribed medications, and ‘yes’ with more than five prescribed medications.^33^

*Psychological parameters*: The Multidimensional Scale for Perceived Social Support (MSPSS)^34^ was used to assess social support. The MSPSS is a brief self-report questionnaire that contains 12 items rated on a five-point Likert-type scale. The MSPSS assesses three subscales of social support: family, friends and significant others. For the MSPSS, the mean scores were calculated as follows: Significant Others Subscale – sum across items 1, 2, 5 and 10, divided by 4; Family Subscale – sum across items 3, 4, 8 and 11, divided by 4; and Friends Subscale – sum across items 6, 7, 9 and 12, divided by 4. The total scale was calculated as the sum of all 12 items, divided by 12. Any mean scale score ranging from 1 to 2.9 was considered low support, a score of 3 to 5 as moderate support and a score from 5.1 to 7 as high support.

The Patient Health Questionnaire-9 (PHQ-9) was used to screen for depression.^35^ From the depression screening with PHQ-9, depression was defined as: moderate depression (12–14), moderately severe depression (15–19) and severe depression (20–27).^35^

The Diabetes Distress Scale (DDS) was used to measure diabetes distress,^36^ a 17-item scale that captures four critical dimensions of distress: emotional burden (5 items), regimen distress (5 items), interpersonal distress (3 items) and physician distress (4 items). For the DDS and its subscales, those with a mean score of less than 2.0 were recorded as ‘no distress’, a mean item score of 2.0–2.9 was considered ‘moderate distress’, while a mean item score > 3.0 was considered ‘high distress’.^36^

### Data analysis

All data were checked for completeness and encoded. The data were then exported to a Microsoft Excel sheet, which was imported into Stata software version 18 for analysis. Descriptive analysis was computed using frequency and proportions for each explanatory categorical variable. Pearson’s Chi-square test was used to determine associations between the presence of hypertension on one hand and the control of blood pressure on the other hand with exposure variables in bivariate analysis.

A multiple logistic regression model that accounted for clustering by health facility was used to identify the independent factors for the occurrence of hypertension. Age, sex, and duration of diabetes were included in the model as *a priori* guided by literature.^37,38^ The multivariable model included *a priori* selected variables and variables with *p*-value < 0.20 in bivariate analysis.^39^ The adjusted odds ratio (aOR) and the corresponding 95% CI were reported. A two-tailed *p*-value of < 0.05 was considered statistically significant in the final model.

For the control of blood pressure, a multilevel multivariable fixed effects logistic regression model was computed to account for clustering (facility). Only participants with a past history of hypertension were considered in the analysis. The multivariable model included variables with *p*-value < 0.20 on bivariate analysis. Adjusted odds ratio and the corresponding 95% CI were reported. A two-tailed *p*-value of < 0.05 was considered statistically significant in the final model.

### Ethical considerations

The study obtained ethical clearance from two universities, one from ethics committees of the Protestant University of Congo (reference number: CEUPC 0067; date: 05 February 2021) and the Human Research Ethics Committee (Medical) of the University of the Witwatersrand (reference number: M210308; date: 26 August 2021). The study was conducted according to the ethical guidelines of the Declaration of Helsinki. The authors obtained permission from the managers of diabetes healthcare centres in Kinshasa to conduct the study. Informed consent was obtained from each participant. Data collection was performed in strict adherence to local coronavirus disease 2019 (COVID-19) regulations. The researcher obtained written informed consent prior to participation. At the respective facilities, data were collected in quiet rooms. The data collected were anonymous and contained no personal information. They were stored in the REDCap software, and only the principal investigator had the password to access all the data. Analyses were performed and published on pooled data.

## Results

A total of 620 participants were enrolled in the study. The mean age of the participants was 60 ± 12 years. Almost two-thirds of the participants were females (66.0% [409/620]), four-fifths being adults aged 50 years and above (81.3% [504/620]). About two-thirds were married (63.4% [393/620]); the majority were from the Kongo ethnic group (81.0% [502/620]), had not reached university (87.9% [545/620]), were unemployed (67.6% [419/620]), residing near the healthcare centre (74.8% [464/620]), had no health insurance (92.1% [571/620]) and lived below the poverty line (76.3% [473/620]). More than half of the study population were on an insulin-based regimen (53.9% [334/620]). A few had more than five medications (1.3% [8/620]) (Table 1). Out of the 215 who were hypertensive, only a small proportion (29.3% [63/215]) was taking medication.

**TABLE 1:** Participants with type 2 diabetes characteristics and presence of hypertension, Kinshasa, 2021–2022, (*N* = 620).

Characteristic	All participants *n*	Hypertensive			No hypertensive			*p*
		*n*	%	95% CI	*n*	%	95% CI	
**Sex**	-	-	-	-	-	-	-	0.198
Male	409	261	63.8	0.19–0.27	148	36.2	0.35–0.47	-
Female	211	144	68.2	0.08–0.15	67	31.8	0.19–0.27	-
**Age (years)**	-	-	-	-	-	-	-	0.033
18–49	116	87	75.0	0.03–0.07	29	25.0	0.10–0.19	-
50+	504	318	63.1	0.25–0.39	186	36.9	0.44–0.56	-
**Marital status**	-	-	-	-	-	-	-	0.070
Single	199	118	59.3	0.10–0.20	81	40.7	0.16–0.26	-
Married	393	266	67.7	0.16–0.27	127	32.3	0.35–0.50	-
Widow	28	21	75.0	0.003–0.04	7	25.0	0.02–0.06	-
**Ethnic group**	-	-	-	-	-	-	-	0.055
Kongo	502	340	67.7	0.21–0.32	162	32.3	0.43–0.63	-
Luba	31	14	45.2	0.02–0.06	17	54.8	0.01–0.04	-
Ngala	36	17	47.2	0.02–0.06	19	52.8	0.01–0.07	-
Swahili	19	13	68.4	0.004–0.03	6	31.6	0.01–0.05	-
Other	32	21	65.6	0.008–0.04	11	34.4	0.02–0.06	-
**Religion**	-	-	-	-	-	-	-	0.098
Catholic	281	168	59.8	0.14–0.24	113	40.2	0.21–0.32	-
Protestant	128	94	73.4	0.04–0.09	34	26.6	0.12–0.18	-
Independent group	167	114	68.3	0.06–0.13	53	31.7	0.13–0.25	-
Jehovah witness	44	29	65.9	0.02–0.04	15	34.0	0.03–0.07	-
**Educational status**	-	-	-	-	-	-	-	0.922
No tertiary	545	355	65.1	0.25–0.39	190	34.9	0.48–0.63	-
Tertiary	75	50	66.7	0.03–0.08	25	33.3	0.06–0.11	-
**Occupation**	-	-	-	-	-	-	-	0.130
Employed	132	95	72.0	0.04–0.10	37	28.0	0.12–0.20	-
Unemployed	419	267	63.7	0.20–0.32	152	36.3	0.35–0.48	-
Other	69	43	62.3	0.03–0.07	26	37.7	0.04–0.11	-
**Distance (km)**	-	-	-	-	-	-	-	0.853
< 5	465	296	65.6	0.20–0.33	155	34.4	0.38–0.55	-
5 or more	155	109	64.5	0.07–0.16	60	35.5	0.13–0.23	-
**Health insurance**	-	-	-	-	-	-	0.017	-
No	571	382	66.9	0.25–0.39	189	33.1	0.02–0.09	-
Yes	49	23	46.9	0.02–0.09	26	53.1	0.02–0.06	-
**Poverty**	-	-	-	-	-	-	-	0.231
No	473	304	64.3	0.41–0.54	169	35.7	0.22–0.36	-
Yes	147	101	68.7	0.13–0.21	46	31.3	0.05–0.12	-
**Duration of diabetes (years)**	-	-	-	-	-	-	-	< 0.001
< 5	267	200	74.9	0.09–0.14	67	25.1	0.25–0.40	-
5 or more	353	205	58.1	0.19–0.32	148	41.9	0.29–0.37	-
**Glycaemic control**	-	-	-	-	-	-	-	0.929
No	200	121	60.5	0.23–0.38	79	39.5	0.43–0.61	-
Yes	420	284	67.6	0.04–0.10	136	32.4	0.08–0.17	-
**Presence of complications**	-	-	-	-	-	-	-	< 0.001
No	219	0	0.0	-	219	100.0	0.26–0.44	-
Yes	401	215	53.6	0.29–0.44	186	46.4	0.29–0.44	-
**Overweight**	-	-	-	-	-	-	-	0.000
No	412	305	74.0	0.12–0.20	107	26.0	0.35–0.54	-
Yes	208	100	48.1	0.14–0.29	108	51.9	0.13–0.27	-
**Depression**	-	-	-	-	-	-	-	0.275
None	612	401	99.0	0.28–0.43	211	98.1	0.55–0.70	-
Moderate	8	3	2.0	0.002–0.03	3	1.9	0.0018–0.0194	-

CI, confidence interval; km, kilometre.

### Hypertension-associated factors

After adjusting for random and fixed effects at facility level in the multivariable logistic model, overweight (aOR = 2.73; 95% CI: 1.79–4.15; *p* = 0.000), diabetes duration (aOR = 2.01; 95% CI: 1.25–2.76; *p* = 0.002), severe diabetes distress (aOR = 0.47; 95% CI: 0.28–0.79; *p* = 0.004), unemployment (aOR = 1.93; 95% CI: 1.18–3.17; *p* = 0.009) and being of the protestant religion (aOR = 0.48; 95% CI: 0.29–0.82; *p* = 0.005) were associated with hypertension (Table 2).

**TABLE 2:** Results of mixed effects logistic regression estimating the odds for presence of hypertension among participants with type 2 diabetes in Kinshasa, 2021–2022.

Characteristic	Univariate analysis			Multivariate analysis		
	*p*	cOR	95% CI	*p*	aOR	95% CI
**Age group (years)**						
18–49	1	1	-	1	1	-
50+	0.016	1.75	1.11–0.77	0.140	1.46	0.88–2.41
**Sex**						
Male	1	1	-	1	1	-
Female	0.272	0.82	0.58–0.69	0.808	1.0	0.70–1.58
**Ethnicity**						
Kongo	0.010	1.71	1.13–0.57	0.888	0.97	0.59–1.58
Other	1	-	-	1	1	-
**Religion**						
Catholic	1	1	-	1	1	-
Protestant	0.008	0.54	0.33–0.85	0.005*	0.48	0.29–0.82
Independent	0.073	0.69	0.46–0.04	0.304	0.79	0.50–1.24
Jehovah witness	0.440	0.77	0.39–0.50	0.220	0.62	0.29–1.33
**Occupation**						
Employed	1	1	-	1	1	-
Unemployed	0.083	1.46	0.95–0.24	0.009*	1.93	1.18–3.17
Other	0.163	1.52	0.84–0.88	0.129	1.73	0.85–3.53
**Overweight**						
No	1	1	-	1	1	-
Yes	0.000	3.08	2.17–0.37	0.000*	2.73	1.79–4.15
**Diabetes distress**						
None	1	1	-	1	-	-
Moderate	0.001	0.48	0.31–0.75	0.124	0.67	0.40–1.12
Severe	0.000	0.34	0.23–0.51	0.004*	0.47	0.28–0.79
**Diabetes duration (months)**						
< 60	1	1	-	1	1	-
> 60	0.000	2.16	1.52–3.05	0.002*	2.01	1.25–2.76
**Glycaemic control**						
Controlled	1	1	-	1	1	-
Uncontrolled	0.082	0.73	0.52–1.04	0.635	0.91	0.61–1.35
**Support**						
Low	1	1	-	1	1	-
Moderate	0.057	1.50	0.99–2.28	0.112	1.48	0.91–2.40
High	0.024	2.92	1.15–7.44	0.058	2.80	0.96–8.17

aOR, adjusted odds ratio; CI, confidence interval; cOR, crude odds ratio.

* , *p*-value is statistically significant at 5ce.

### Blood pressure control and associated factors

From all the participants, 67.6% (419/620) had uncontrolled blood pressure, while for those participants considered as hypertensive, 50.2% (108/215) had uncontrolled hypertension (Table 3).

**TABLE 3:** Participants with type 2 diabetes and control of blood pressure, Kinshasa, 2021–2022, (*n* = 215).

Characteristic	All participants		Controlled blood pressure			Uncontrolled blood pressure			*p*
	*n*	%	*n*	%	95% CI	*n*	%	95% CI	
**Age group (years)**	-	-	-	-	-	-	-	-	0.941
18–49	29	13.4	15	51.7	0.05–0.15	14	48.3	0.04–0.15	-
50+	186	86.5	92	49.5	0.04–0.15	94	50.5	0.28–0.50
**Sex**	-	-	-	-	-	-	-	-	0.44
Male	148	68.8	73	49.3	0.24–0.45	75	50.7	0.14–0.27
Female	67	31.2	34	50.7	0.24–0.40	33	49.3	0.10–0.22
**Ethnicity**	-	-	-	-	-	-	-	-	0.663
Kongo	162	75.3	82	50.6	0.33–0.56	80	49.4	0.06–0.13	-
Other	53	24.7	25	47.2	0.27–0.52	28	52.8	0.05–0.14	-
**Overweight**	-	-	-	-	-	-	-	-	0.003
No	107	49.8	65	60.7	0.26–0.51	42	39.3	0.11–0.21	-
Yes	108	50.2	42	38.9	0.15–0.32	66	61.1	0.16–0.35	-
**Hypertension treatment**	-	-	-	-	-	-	-	-	0.012
No	152	70.7	84	55.2	0.35–0.63	68	44.7	0.02–0.12	-
Yes	63	29.3	23	36.5	0.27–0.52	40	63.5	0.04–0.16	-
**Glucose control**	-	-	-	-	-	-	-	-	0.865
Controlled	79	36.7	33	41.8	0.33–0.57	46	58.2	0.04–0.16	-
Uncontrolled	136	63.3	74	54.4	0.29–0.50	62	45.6	0.03–0.15	-
**Poly prescription**	-	-	-	-	-	-	-	-	0.239
No	208	96.7	105	50.5	0.40–0.65	103	49.5	0.001–0.23	-
Yes	7	3.3	2	28.6	0.33–0.58	3	71.4	0.003–0.03	-
**Duration of diabetes (years)**	-	-	-	-	-	-	-	-	0.623
< 5	67	31.2	35	52.2	0.13–0.31	32	47.8	0.25–0.42	-
5 or more	148	68.8	72	48.6	0.10–0.23	76	51.4	0.22–0.41	-
**Support**	-	-	-	-	-	-	-	-	0.180
Low	38	17.7	23	60.5	0.08–0.22	15	39.5	0.30–0.50	-
Moderate	177	82.3	84	47.5	0.04–0.13	93	52.5	0.28–0.52	-

CI, confidence interval.

After adjusting for random and fixed effects at facility level in the multivariable logistic model, only overweight remained a significant determinant of uncontrolled hypertension. The odds of having uncontrolled blood pressure were significantly higher among overweight diabetic patients (aOR=2.02; 95% CI: 1.08–3.79; *p* = 0.028; Table 4).

**TABLE 4:** Factors associated with uncontrolled blood pressure.

Characteristic	Univariate analysis			Multivariate analysis		
	*p*	cOR	95% CI	*p*	aOR	95% CI
**Age group**						
18–49	1	1	-	1	1	-
50+	0.821	1.09	0.50–2.40	0.844	0.92	0.38–2.20
**Sex**						
Male	1	1	-	1	1	-
Female	0.847	0.94	0.53–1.68	0.868	1.06	0.56–1.99
**Ethnicity**						
Kongo	0.663	1.15	0.62–2.14	-	-	-
Other	1	-	-	-	-	-
**Overweight**						
No	1	1	-	1	1	-
Yes	0.001	2.43	1.41–4.21	0.028*	2.02	1.08–3.79
**Hypertension treatment**						
No	1	1	-	1	1	-
Yes	0.013	2.15	1.17–3.93	0.194	1.62	0.78–3.37
**Glucose control**						
Controlled	1	1	-	1	1	-
Uncontrolled	0.075	0.60	0.34–1.05	0.346	1.34	0.73–2.48
**Poly prescription**						
No	1	1	-	1	1	-
Yes	0.270	2.55	0.48–13.43	0.752	1.34	0.22–8.00
**Duration of diabetes**						
< 5 years	1	1	-	1	1	-
5 years and more	0.626	1.15	0.65–2.06	0.841	1.07	0.55–2.08
**Support**						
Low	1	1	-	1	1	-
Moderate	0.146	1.70	0.83–3.47	0.392	1.40	0.64–3.08

cOR, crude odds ratio; CI, confidence interval; aOR, adjusted odds ratio.

## Discussion

The study sought to determine the prevalence of hypertension and that of uncontrolled hypertension and their respective associated factors among patients with T2D in a primary care setting in Kinshasa, DRC. The study showed that more than one-third of T2D patients had hypertension and among those hypertensives over half had uncontrolled hypertension. Being overweight, unemployment and longer duration of diabetes were associated with increased odds of hypertension while having severe diabetes distress and being of the protestant religion decreased odds of hypertension. In terms of blood pressure control, only overweight was found to be significantly associated with an increased risk of uncontrolled hypertension.

The prevalence of hypertension in this study was consistent with findings from other settings. For instance, a study conducted in Ethiopia found that the prevalence of hypertension was 37.4%.^37^ Nonetheless, findings from this study pointed to a reduced prevalence in comparison to a systematic review that aimed at measuring the regional prevalence of hypertension that reported more than half (58.1%) of people with T2DM in Africa are hypertensive such as South Africa (92%), Ethiopia (84.9%), Zimbabwe (80%) and Ghana (74.6%).^10^ Possible reasons for such a reduction might be the differences in the study population, study setting and difference in the lifestyle of participants. Available evidence suggests that hypertension is a growing health problem in DRC. This finding also pointed out an increasing burden of noncommunicable diseases (NCDs) in the African region, suggesting a need to strengthen health systems to be able to develop interventions to tackle this burden. In Kinshasa, the prevalence of the hypertension among patients with T2D calls for the strengthened integration of the World Health Organization (WHO) package of essential noncommunicable (PEN) diseases in primary health care in low-resource settings.^40^

Sociodemographic factors such as unemployment and religious affiliation were significantly associated with hypertension. Unemployment emerged as a significant risk factor, aligning with previous research that indicates financial stress associated with unemployment contributes to poor health outcomes, including hypertension.^41^ However, contrary to these findings, some evidence shows that being employed is associated with the development of hypertension among T2DM patients.^10^ In Kinshasa, patients with diabetes could benefit from the alleviation of the financial burden through the extension of the universal coverage to NCDs.^42^ Currently, in Kinshasa, only maternity and neonatal care are available at no cost to the patients.

This study found that being of the protestant affiliation was associated with decreased odds of hypertension. The available literature has conflicting findings on the effects of religion on chronic conditions such as diabetes and hypertension.^43,44^ Overall, religious practices (higher religiosity) have been part of the coping mechanisms for illnesses.^43,44^ Some studies suggested that religious involvement and associated social support could contribute to better health outcomes, including lower blood pressure.^45^

Among the clinical characteristics, the duration of diabetes and being overweight were significantly associated with hypertension. The duration of diabetes was the sole nonmodifiable factor identified. Patients diagnosed with diabetes for more than 5 years had nearly twice the odds of developing hypertension compared to those diagnosed within the last 5 years. This finding aligns with the literature suggesting that the duration of diabetes significantly influences hypertension development among type 2 diabetic patients.^10,46^ However, some studies reported heterogeneous findings on the association of duration of diabetes and hypertension.^47^

Consistent with previous studies, overweight was found to be associated with hypertension.^10,48,49,50^ Attaining and maintaining a healthy weight improves blood pressure and diabetes management and reduces cholesterol levels. The mechanism behind this association is multifaceted, involving insulin resistance, increased sympathetic nervous activity and altered renal function.^51^ Emphasis on weight management has been identified as a priority as it has been pointed out as a leading cause of many disorders such as diabetes and hypertension.^52^ However, some studies have suggested that T2D may occur because of metabolic syndrome even with a normal BMI and waist circumference.^52^ This finding points out the importance of weight management in achieving blood pressure control among hypertensive patients, as supported by numerous clinical guidelines.^53^

Our study found that participants with high diabetes distress had decreased odds of having hypertension. Our finding was in discordance with what is usually described in the literature.^54^ It can be hypothesised that there were some coping strategies among the participants, such as religious practices, which reduced the known deleterious effect of diabetes-related distress and prevented it from leading to hypertension.

The study identified a high prevalence of uncontrolled hypertension among T2D patients, with over half (50.2%) of the participants having uncontrolled hypertension. This uncontrolled hypertension could be attributed to the fact that there was less than one-third of hypertensive patients who were on hypertension treatment in this study. This prevalence is consistent with other studies conducted in Africa and beyond, such as Uganda (82.5%),^21^ Malaysia (57%)^17^ and Iran (58.7%).^16^ A systematic review conducted in SSA reported that prevalence of uncontrolled hypertension among T2DM ranged from 54% to 85%.^19^ The WHO’s global target on hypertension control action plan recommends integrated care programmes for the management of hypertension and comorbidities.^55^

Overweight was the only significant factor contributing to uncontrolled hypertension among patients with T2DM in Kinshasa, and these findings are consistent with findings from other settings.^16,56^ Therefore, addressing overweight through lifestyle modifications and adherence to diabetes care guidelines is crucial in managing hypertension in T2DM patients.

### Limitations and strengths of the study

The cross-sectional nature of the study is unable to draw a definitive conclusion on the relationships found in the study regarding the presence of hypertension among patients with T2D on one hand and uncontrolled hypertension on the other hand. Some of the information sought from the participants was sensitive; our findings could have been influenced by social desirability bias. We sought to minimise this bias by training the data collectors to be nonjudgemental. The sampling method used enhanced the generalisation of the study findings to the patients with T2D in Kinshasa Primary Care Network and similar settings in DRC and SSA.

Beyond these limitations, this study provided an overview of the epidemiology of hypertension and its control among patients with T2D in Kinshasa, DRC. However, future longitudinal studies could further explore the drivers of hypertension and uncontrolled hypertension among diabetes patients in Kinshasa and other similar settings.

## Conclusion

This study in Kinshasa, DRC, investigated the epidemiology of hypertension and uncontrolled hypertension among patients with T2D. The findings revealed that about one-third of participants with T2D were hypertensive and half of them had uncontrolled hypertension. Being overweight was the sole nonmodifiable factor related to the risk of hypertension and that of uncontrolled hypertension. There is a need to strengthen lifestyle modifications as part of hypertension management among patients with T2D. Particularly, the adoption of cost-effective interventions in our setting and tackling weight gain must be encouraged among patients with T2D. There is need to strengthen the healthcare system for diabetes care through the integration of new approaches for better management and more active participation of the patients with diabetes through better education and motivation.
